# The Immune Receptor Roq1 Confers Resistance to the Bacterial Pathogens *Xanthomonas*, *Pseudomonas syringae*, and *Ralstonia* in Tomato

**DOI:** 10.3389/fpls.2020.00463

**Published:** 2020-04-23

**Authors:** Nicholas C. Thomas, Connor G. Hendrich, Upinder S. Gill, Caitilyn Allen, Samuel F. Hutton, Alex Schultink

**Affiliations:** ^1^Fortiphyte Inc., Berkeley, CA, United States; ^2^Innovative Genomics Institute, University of California, Berkeley, Berkeley, CA, United States; ^3^Department of Plant Pathology, University of Wisconsin–Madison, Madison, WI, United States; ^4^IFAS, Gulf Coast Research and Education Center, University of Florida, Wimauma, FL, United States; ^5^Department of Plant Pathology, North Dakota State University, Fargo, ND, United States

**Keywords:** plant immunity, *Ralstonia*, *Xanthomonas*, *Pseudomonas*, tomato

## Abstract

*Xanthomonas* species, *Pseudomonas syringae* and *Ralstonia* species are bacterial plant pathogens that cause significant yield loss in many crop species. Generating disease-resistant crop varieties can provide a more sustainable solution to control yield loss compared to chemical methods. Plant immune receptors encoded by nucleotide−binding, leucine−rich repeat (NLR) genes typically confer resistance to pathogens that produce a cognate elicitor, often an effector protein secreted by the pathogen to promote virulence. The diverse sequence and presence/absence variation of pathogen effector proteins within and between pathogen species usually limits the utility of a single NLR gene to protecting a plant from a single pathogen species or particular strains. The NLR protein Recognition of XopQ 1 (Roq1) was recently identified from the plant *Nicotiana benthamiana* and mediates perception of the effector proteins XopQ and HopQ1 from *Xanthomonas* and *P. syringae* respectively. Unlike most recognized effectors, alleles of XopQ/HopQ1 are highly conserved and present in most plant pathogenic strains of *Xanthomonas* and *P. syringae*. A homolog of XopQ/HopQ1, named RipB, is present in most *Ralstonia* strains. We found that Roq1 confers immunity to *Xanthomonas*, *P. syringae*, and *Ralstonia* when expressed in tomato. Strong resistance to *Xanthomonas perforans* was observed in three seasons of field trials with both natural and artificial inoculation. The *Roq1* gene can therefore be used to provide safe, economical, and effective control of these pathogens in tomato and other crop species and reduce or eliminate the need for traditional chemical controls.

## Introduction

Bacterial pathogens from the species *Pseudomonas syringae* and the genera *Ralstonia* and *Xanthomonas* can infect many different crop species and inflict significant yield losses when environmental conditions favor disease. *Xanthomonas* and *P. syringae* tend to enter plant stem, leaf, or flower tissue through wounds or natural openings, such as stomata or hydathodes, whereas *Ralstonia* is soilborne, entering roots through wounds and natural openings before colonizing xylem tissue (Vasse et al., 1995; Gudesblat et al., 2009). Once inside the host these bacteria manipulate host metabolism and suppress plant immunity using multiple strategies, including effector proteins delivered by the type III secretion system (Kay and Bonas, 2009; Peeters et al., 2013; Xin et al., 2018). This enables the pathogens to multiply to high titers while the plant tissue is still alive and showing few or no visual symptoms. Once the bacteria reach high populations, they typically cause necrosis of infected leaf tissue or wilting and eventual death of the plant.

Effective control measures for bacterial pathogens are relatively limited, particularly once plants become infected (Davis et al., 2013). Soil fumigation can reduce *Ralstonia* populations in the soil but this is expensive, potentially hazardous to workers and the environment, and of limited efficacy (Yuliar et al., 2015). Copper sulfate and antibiotics such as streptomycin have been used to control *Xanthomonas* species and *P. syringae* but have adverse environmental impacts and many strains have evolved tolerance to these chemicals (Kennelly et al., 2007; Griffin et al., 2017). Applying chemicals that induce systemic acquired resistance, such as acibenzolar-S-methyl, can provide partial control but increases production cost and can depress crop yields when used repeatedly (de Pontes et al., 2016).

The most effective, economical, and safe way to control bacterial pathogens is to plant crop varieties that are immune to the target pathogen (Jones et al., 2014; Vincelli, 2016). Such immunity is often mediated by plant immune receptor genes. Plants have large families of cell surface and intracellular immune receptor proteins that surveil for the presence of invading pathogens (Zipfel, 2014; Jones et al., 2016). Effector proteins delivered by the bacterial type III secretion system are common elicitors of intracellular plant immune receptors encoded by nucleotide−binding domain and leucine−rich repeat containing (NLR) genes (Li et al., 2015; Jones et al., 2016; Kapos et al., 2019). While effector proteins contribute to virulence on a susceptible host, an immune response is activated in the plant if that plant has the cognate receptor to recognize the effector. NLR genes typically confer strong, dominant resistance to pathogens that deliver the cognate recognized effector protein (Tai et al., 1999; Jones and Dangl, 2006; Boller and He, 2009; Deslandes and Rivas, 2012; Li et al., 2015). Disease-resistant plants can be generated by identifying the appropriate plant immune receptor genes and transferring them into the target crop species (Dangl et al., 2013).

We recently identified the *Nicotiana benthamiana* immune receptor gene named *Recognition of XopQ 1* (*Roq1*), which appears to be restricted to the genus *Nicotiana* and contributes to resistance against *Xanthomonas* spp. and *P. syringae* (Schultink et al., 2017). The Roq1 protein is a Toll/Interleukin−1 Receptor (TIR) NLR immune receptor that mediates recognition of the *Xanthomonas* effector protein XopQ and the homologous effector HopQ1 from *P. syringae*. XopQ is present in most species and strains of *Xanthomonas* (Ryan et al., 2011) and HopQ1 is present in 62% (290 of 467) sequenced putative pathogenic *P. syringae* strains (Dillon et al., 2019). XopQ/HopQ1 has homology to nucleoside hydrolases and has been shown to enhance virulence on susceptible hosts (Ferrante and Scortichini, 2009; Li et al., 2013), possibly by altering cytokinin levels or interfering with the activity of host 14-3-3 proteins (Giska et al., 2013; Li et al., 2013; Hann et al., 2014; Teper et al., 2014). The conservation of XopQ/HopQ1 and their importance in virulence suggests that *Roq1* has widespread potential to confer resistance to these pathogens in diverse crop species. Indeed, transient expression assays demonstrated that *Roq1* can recognize XopQ/HopQ1 alleles from *Xanthomonas* and *P. syringae* pathogens of tomato, pepper, rice, citrus, cassava, brassica, and bean (Schultink et al., 2017). However, it was not known if *Roq1* can confer disease resistance when expressed in a crop plant.

Tomato is one of the most important vegetable crops and is highly susceptible to several bacterial diseases. Bacterial spot, bacterial speck, and bacterial wilt of tomato are caused by *Xanthomonas* species, *P. syringae* pv. *tomato* and *Ralstonia*, respectively. These diseases are difficult to control, especially if the pathogens become established in a field and environmental conditions favor disease (Rivard et al., 2012; Potnis et al., 2015). Tomato breeding germplasm has only limited resistance against these diseases and in some cases linkage drag has complicated introgression of resistance genes from wild relatives (Sharma and Bhattarai, 2019). *Ralstonia* contains a homolog of XopQ/HopQ1 called RipB. Roq1 is able to mediate the perception of RipB (Staskawicz and Schultink, 2019), and silencing *Roq1* in *N. benthamiana* resulted in severe wilting phenotypes upon *Ralstonia* infection (Nakano and Mukaihara, 2019). This suggests that expressing *Roq1* in tomato could also confer resistance to bacterial wilt. Like XopQ/HopQ1 in *Xanthomonas* and *P. syringae*, RipB is highly conserved and is present in approximately 90% of sequenced *Ralstonia* isolates (Sabbagh et al., 2019). Here we present data showing that expression of *Roq1* in tomato confers resistance against *Xanthomonas*, *Pseudomonas*, and *Ralstonia* upon recognition of the cognate pathogen effector.

## Materials and Methods

### Generation of Tomato Expressing Roq1

The Roq1 coding sequence was amplified from *N. benthamiana* cDNA and cloned into the pORE E4 binary plasmid (Coutu et al., 2007). The expression of *Roq1* was driven by the constitutive P_ENTCUP__2_ promoter, which was derived from tobacco and has been reported to drive expression in leaf, root, and stem tissue (Malik et al., 2002). *Agrobacterium tumefaciens* co-cultivation was used to transform *Roq1* into the tomato variety Fla. 8000 at the University of Nebraska Plant Transformation Core Research Facility. Transformed plants were selected by resistance to kanamycin, confirmed by genotyping, and selfed to obtain homozygous lines.

### Bacterial Leaf Spot and Leaf Speck Disease Assays

*Xanthomonas* cultures were grown in NYG broth (0.5% peptone, 0.3% yeast extract, 2% glycerol) with rifampicin (100 μg/mL) overnight at 30°C. *P. syringae* cultures were grown in KB broth (1% peptone, 0.15% K_2_HPO_4_, 1.5% glycerol, 5 mM MgSO_4_, pH 7.0) with rifampicin (100 μg/mL) overnight at 28°C. Bacterial cultures were spun down at 5200 *g*, washed once with 10 mM MgCl_2_, and then diluted to the appropriate infiltration density with 10 mM MgCl_2_. Leaf tissue of tomato plants (approximately 4 weeks old) was infiltrated with bacterial solution using a needleless syringe. To quantify bacterial growth, leaf punches were homogenized in water, serially diluted and plated on NYG (for *Xanthomonas* spp.) or KB (for *P. syringae*) plates supplemented with 100 μg/mL rifampicin and 50 μg/mL cycloheximide to measure colony forming units. Three biological replicates were performed for each condition and the reported results are representative of at least three independent experiments. *Xanthomonas perforans* strain 4B, *Xanthomonas euvesicatoria* strain 85-10, and *P. syringae* strain DC3000 and the corresponding XopQ/HopQ1 deletion mutants and complemented strains were described previously (Wei et al., 2007; Schwartz et al., 2015; Schultink et al., 2017). The *P. syringae* pv. *tomato* Race 1 strain was isolated from a field of tomatoes with the PTO resistance gene in 1993 in California.

### Transient Expression of RipB and XopQ

Alleles of RipB from *Ralstonia* strains GMI1000 and MolK2 (NCBI Genbank accessions CAD13773.2 and WP_003278485) were synthesized and cloned into a *Bsa*I-compatible version of the pORE E4 vector (Coutu et al., 2007). This plasmid was transformed into *A. tumefaciens* strain C58C1. *A. tumefaciens* cultures were grown on a shaker overnight at 30°C in LB broth with rifampicin (100 μg/mL), tetracycline (10 μg/mL), and kanamycin (50 μg/mL). The cells were collected by centrifugation and resuspended in infiltration buffer [10 mM 2-(*N*-morpholino)ethanesulfonic acid, 10 mM MgCl_2_, pH 5.6], and diluted to an OD_600_ of 0.5 for infiltration into *Nicotiana tabacum* leaf tissue. Each experiment was performed on multiple leaves and multiple plants with the selected images being representative of the observed result.

### *N. tabacum roq1* Mutant Lines

*Nicotiana tabacum roq1* mutant lines were generated by transforming *N. tabacum* with a construct coding for CAS9 and a guide RNA targeting the *Roq1* gene with the sequence GATGATAAGGAGTTAAAGAG. This construct was also used for the generation of *N. benthamiana roq1* mutants published in Qi et al. (2018). Transformed *N. tabacum* plants were generated by *Agrobacterium* co-cultivation and selected for using kanamycin. Transformed plants were genotyped for the presence of mutations at the target site by PCR and Sanger sequencing (Supplementary Table S1).

### Bacterial Wilt Virulence Assays

*Ralstonia* virulence on tomato was measured as previously described (Khokhani et al., 2018). Briefly, cells of *Ralstonia* strains GMI1000 and UW551 grown overnight in CPG (0.1% casein hydrolysate, 1% peptone, 0.5% glucose, pH 7.0) at 28°C were collected by centrifugation and diluted to an OD_600_ of 0.1 in water (1 × 10^8^ CFU/mL). 50 mL of this suspension was poured on the soil around 17-day old tomato plants. Disease was rated daily for two weeks on a 0–4 disease index scale, where 0 is no leaves wilted, 1 is 1–25% wilted, 2 is 26–50% wilted, 3 is 51–75% wilted, and 4 is 76–100% wilted. Data represent a total of four biological replicates with 10 plants per replicate. Virulence data were analyzed using repeated measures ANOVA (Khokhani et al., 2018). For petiole infection, the petiole of the first true leaf was cut with a razor blade horizontally approximately 1 cm from the stem. A drop of bacterial solution (2 μL, OD_600_ = 0.001) was pipetted onto the exposed cut petiole surface.

### Field Trial Disease Assays

**FIGURE 1:**
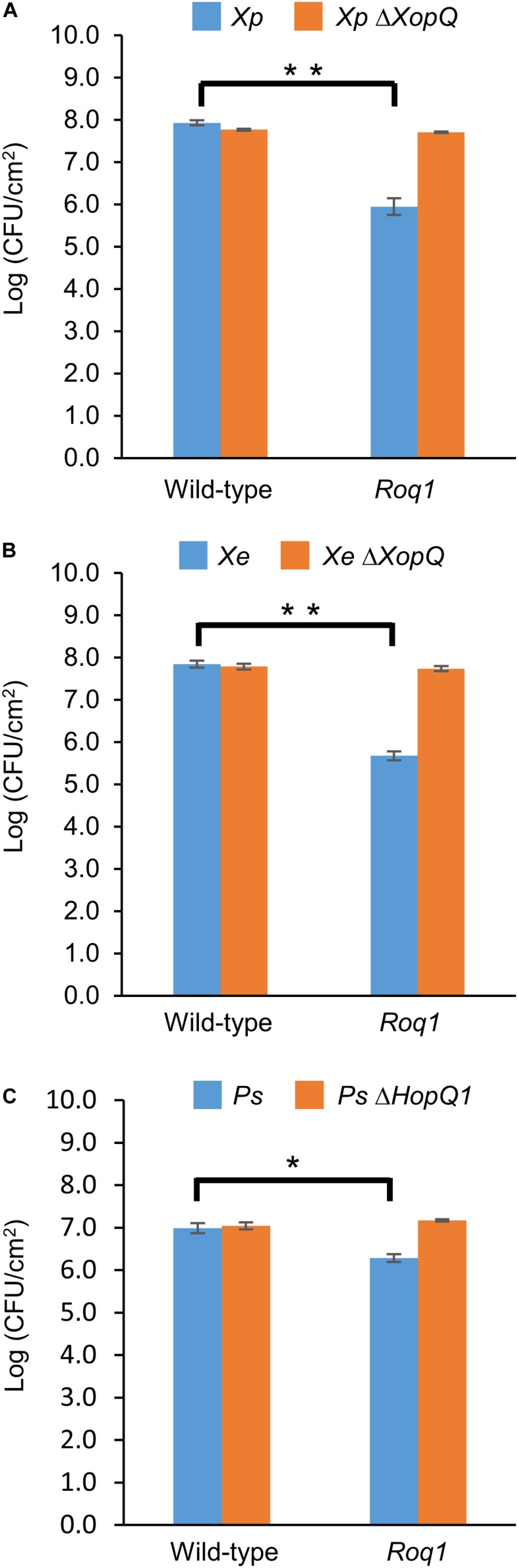
Bacterial growth in tomatoes expressing Roq1. *Xanthomonas perforans* 4B (*Xp*), *Xanthomonas euvesicatoria* 85-10 (*Xe*), and *Pseudomonas syringae* DC3000 (*Ps*) were infiltrated into leaf tissue of wild-type tomato and tomato expressing Roq1 at a low inoculum (OD_600_ = 0.0001 for *Xe* and *Xp*; OD_600_ = 0.00005 for *Ps*). Bacterial abundance was quantified by homogenizing leaf punches and counting colony forming units (CFU) per square centimeter of leaf tissue at 6 days post infiltration for *Xe* and *Xp*; 3 days post infiltration for *Ps*. Error bars indicate standard deviation from three biological replicates. *p < 0.05, **p < 0.01 by Student’s *t*-test.

**FIGURE 2:**
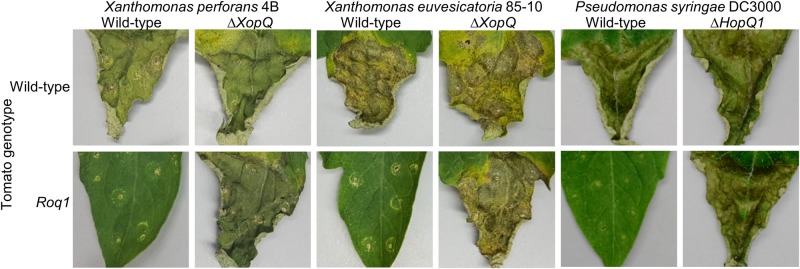
Bacterial disease symptoms on *Roq1* tomato. *Xanthomonas perforans* 4B (*Xp*), *Xanthomonas euvesicatoria* 85-10 (*Xe*), and *Pseudomonas syringae* DC3000 (*Ps*) wild-type and XopQ/HopQ1 knockout strains were infiltrated into tomato leaf tissue at low inoculum and disease symptoms were imaged at 12, 13, and 4 days post infiltration for *Xe*, *Xp*, and *Ps*, respectively. The infiltration was performed using a needleless syringe and circular wounds from the infiltration are visible. The distal part of region of each leaf was infiltrated and the proximal part was left untreated. *Xe* and *Xp* were infiltrated at an OD_600_ of 0.0001 whereas *Ps* was infiltrated at an OD_600_ of 0.00005. The images shown here are representative of at least three independent experiments.

Three field trials were conducted at the University of Florida Gulf Coast Research and Education Center in Balm during the spring seasons of 2018 and 2019 and the fall season of 2018 and under the notification process of the United States Department of Agriculture. Large-fruited, fresh market tomato lines were used in these trials and included the inbred line, Fla. 8000, and nearly isogenic lines containing either *Roq1* (event 316.4) or *Bs2* (Kunwar et al., 2018). The *Roq1* tomato line selected for the field trial was the same line used in the experiments shown in Figures 1, 2, 5. For each trial, seeds were sown directly into peat-lite soilless media (Speedling, Sun City, FL, United States) in 128-cell trays (38 cm^3^ cell size). Transplants were grown in a greenhouse until 5 or 6 weeks, then planted to field beds that had been fumigated and covered with reflective plastic mulch. Field trials were conducted using a randomized complete block design with four blocks and 10-plant plots. Field plants were staked and tied, and irrigation was applied through drip tape beneath the plastic mulch of each bed. A recommended fertilizer and pesticide program were followed throughout the growing season, excluding the use of plant defense inducers, copper, or other bactericides (Freeman et al., 2018). Fruits were harvested from the inner eight plants of each plot at the breaker stage and beyond and graded for marketability according to USDA specifications with block considered a random effect.

Field trials were inoculated with *X. perforans* race T4 (strain mixture of GEV904, GEV917, GEV1001, and GEV1063). Bacterial strains were grown on nutrient agar medium (BBL, Becton Dickinson and Co., Cockeysville, MD, United States) and incubated at 28°C for 24 h. Bacterial cells were removed from the plates and suspended in a 10 mM MgSO_4_ solution, and the suspension was adjusted to OD_600_ = 0.3, which corresponds to 10^8^ CFU/mL. The suspension for each strain was then diluted to 10^6^ CFU/mL, mixed in equal volume, and applied along with polyoxyethylene sorbitan monolaurate (Tween 20; 0.05% [vol/vol]) for field inoculation. Field trial plants were inoculated approximately 3 weeks after transplanting.

Bacterial spot disease severity was recorded three to eight weeks after inoculation using the Horsfall-Barratt scale (Horsfall and Barrat, 1945), and ratings were converted to midpoint percentages for statistical analysis. Blocks were considered random effects.

### Generation of the *Ralstonia ΔripB* Mutant

An unmarked Δ*ripB* mutant was created using *sacB* selection with the vector pUFR80 (Castañeda et al., 2005). Briefly, the regions upstream and downstream of *ripB* were amplified using the primers ripBupF/R and ripBdwnF/R (Supplementary Table S1). These fragments were inserted into pUFR80 digested with *Hin*dIII and *Eco*RI using Gibson Assembly (Gibson et al., 2009) (New England Biolabs, Ipswitch, MA, United States) and this construct was incorporated into the genome of strain GMI1000 using natural transformation, with successful integrants selected on CPG + kanamycin (Coupat et al., 2008). Plasmid loss was then selected for on CPG plates containing 5% w/v sucrose. Correct deletions were confirmed using PCR and sequencing.

### Phylogenetic Analysis of XopQ, HopQ1, and RipB Alleles

RipB alleles were identified by BLAST search of the NCBI protein database. Clustal Omega (Sievers et al., 2011) was used to generate a multiple sequence alignment with XopQ and HopQ1 alleles. To span the diversity of RipB alleles without having many redundant sequences, only a single sequence was retained if there were multiple identical or nearly identical sequences identified. A maximum likelihood tree was generated using PhyML (Guindon et al., 2010). The phylotype calls of the strains were obtained from previously published worked (Liu et al., 2009; Mukaihara and Tamura, 2009; Safni et al., 2014).

## Results

### Tomatoes Expressing *Roq1* Are Resistant to *Xanthomonas* and *P. syringae*

We generated homozygous tomato plants expressing the *Roq1* gene from *N. benthamiana* and tested them for resistance to *Xanthomonas* and *P. syringae* by measuring bacterial growth *in planta*. Population sizes of wild-type *X. perforans* strain 4B and *X. euvesicatoria* strain 85-10 were approximately 100-fold smaller in tomato expressing *Roq1* compared to wild-type tomato at 6 days post inoculation (Figure 1). In contrast, XopQ deletion mutants multiplied equally well in leaves of both wild-type and *Roq1* tomato. Disease symptoms begin as small water-soaked lesions and progress to necrosis of infected tissue. Wild-type *X. perforans* and *X. euvesicatoria* caused severe disease symptoms on wild-type tomato plants but failed to cause visible symptoms on *Roq1* plants (Figure 2). The XopQ mutants caused similar disease symptoms on both wild-type and *Roq1* tomato. Similar results were observed for *P. syringae* DC3000, and its HopQ1 mutant (Figures 1, 2) and a Race 1 isolate of *P. syringae* pv. *tomato* (Supplementary Figure S1). Tomatoes expressing *Roq1* were resistant to *Xanthomonas* and *Pseudomonas* XopQ/HopQ1 mutants complemented with a wild-type copy of XopQ/HopQ1 (Supplementary Figure S2). A second tomato line expressing *Roq1*, derived from an independent transformation event, also showed resistance to wild-type *X. euvesicatoria* and *X. perforans* but not to the XopQ deletion strains (Supplementary Figure S3).

### Expression of *Roq1* Confers Resistance to *Xanthomonas perforans* in the Field

To determine if the resistance observed in growth chamber experiments would hold up under commercial tomato production conditions, we tested the ability of *Roq1* tomatoes to resist *X. perforans* infection in the field. *Roq1* tomatoes were grown along with the Fla. 8000 wild-type parent as well as a Fla. 8000 variety expressing the *Bs2* gene from pepper as a resistant control (Kunwar et al., 2018). For each of the three growing seasons, both *Roq1* and the resistant *Bs2* control tomatoes showed significantly lower disease severity than the parental Fla. 8000 variety (Table 1) (*p* < 0.05). The total marketable yield of the *Roq1* plants was not significantly different from that of the susceptible parent for any of the three seasons (*p* > 0.05). No obvious difference in growth morphology was observed between *Roq1* and wild-type tomato plants (Supplementary Figure S4).

**TABLE 1:** Field trial results.

**Season/Genotype**	**Disease severity**	**Marketable yield (kg/ha)**
**Spring 2018**		
Fla. 8000	865	54,6559,450
Fla. 8000 Roq1	11	52,6563,810
Fla. 8000 Bs2	11	66,27010,309
**Fall 2018**		
Fla. 8000	257	19,57611,038
Fla. 8000 Roq1	51	18,5385,901
Fla. 8000 Bs2	00	33,77013,176
**Spring 2019**		
Fla. 8000	842	73,00915,243
Fla. 8000 Roq1	117	92,83711,072
Fla. 8000 Bs2	51	80,51614,531

*A field trial was conducted in Florida with disease pressure from *Xanthomonas perforans*. Disease severity presented as percent infected tissue, converted from field ratings that were scored using the Horsfall–Barratt scale. Harvested tomatoes were graded and sized by USDA specifications to calculate the total marketable yield. The values shown are means ± standard deviation from at least four replicate plots of 10 plants each. Tomato plants expressing the Bs2 immune receptor gene were included as a resistant control.*

### The *Ralstonia* Effector RipB, a Homolog of XopQ/HopQ1, Is Recognized by Roq1

**FIGURE 3:**
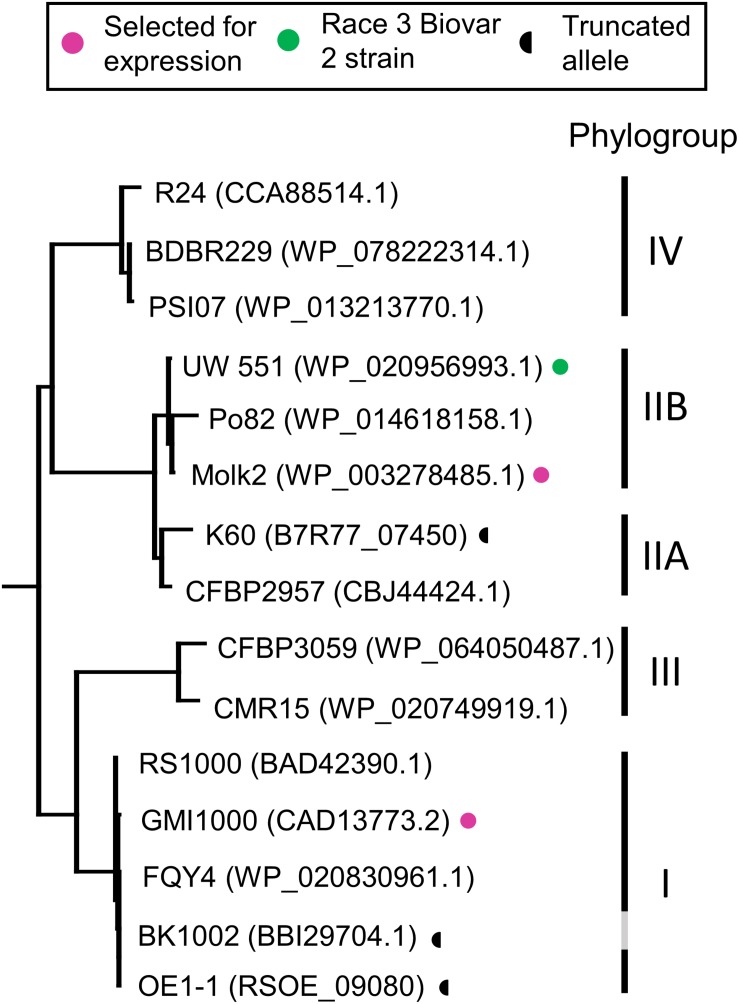
Phylogenetic tree of RipB proteins. RipB alleles from *Ralstonia* strains GMI1000 and MolK2 were cloned for testing in this study and are indicated by magenta dots. Putatively non-functional RipB alleles with C-terminal truncations are marked with a half circle. Strain names are given along with the associated NCBI protein accession or locus tag. Phylotype groups were labeled based on previous publications with the exception of BK1002, for which an assignment was not available. The tree was rooted using XopQ and HopQ1.

**FIGURE 4:**
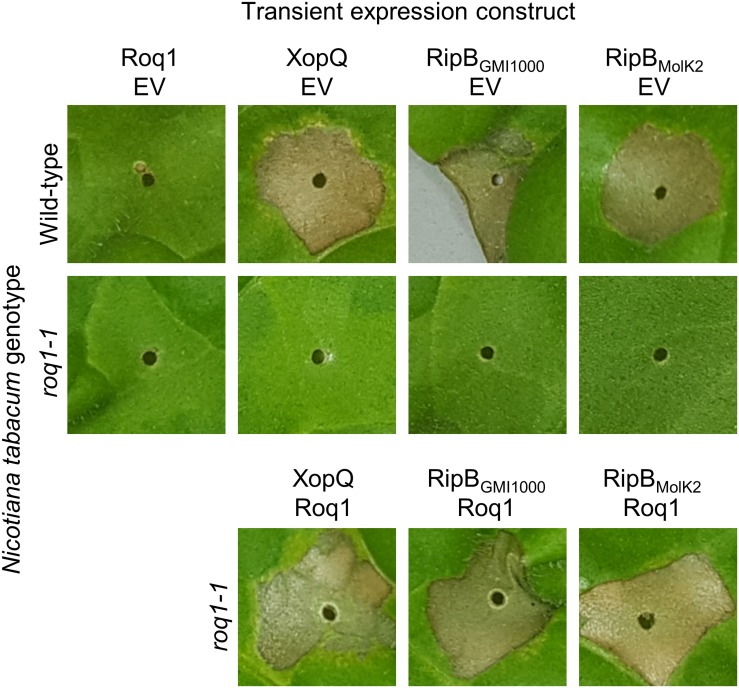
Roq1-dependent recognition of RipB in *Nicotiana tabacum*. *Agrobacterium tumefaciens* was used to transiently express XopQ, RipB_GMI__1000_ and RipB_Molk__2_ along with either Roq1 or an empty vector (EV) control in wild-type *Nicotiana tabacum* and a *roq1* loss of function mutant. The *Agrobacterium* was infiltrated at a total OD_600_ of 0.5 and the leaves were imaged at 3 days post infiltration. The images shown here are representative of three independent experiments.

RipB, considered a “core” *Ralstonia* effector, is present in approximately 90% of sequenced strains (Sabbagh et al., 2019) making it an attractive target ligand for engineering crop plants to be resistant to this pathogen. Roq1 perceives diverse alleles of XopQ and HopQ1 and we hypothesized that it can also recognize different alleles of RipB. We constructed a phylogenetic tree using a subset of RipB alleles identified by BLAST search to approximately span the diversity of this effector in *Ralstonia* (Figure 3). The *Ralstonia* genus contains many diverse strains that have been divided into four phylotypes based on based on sequence analysis of the internal transcribed spacer region of the 16S–23S rRNA gene (Poussier et al., 2000; Prior and Fegan, 2004; Safni et al., 2014). We selected RipB alleles from *Ralstonia* strains GMI1000 and MolK2, from phylotypes I and II, respectively, for subsequent analysis. These two RipB alleles share 71% amino acid identity with each other and approximately 52% identity with XopQ excluding the divergent N terminus containing the putative type III secretion signal. An alignment of these two RipB proteins with XopQ and HopQ1 is shown in Supplementary Figure S5. To test for Roq1-dependent recognition of RipB, we used *Agrobacterium* to transiently express RipB from GMI1000 and Molk2 in leaf tissue of wild-type and *roq1* mutant *N. tabacum*. The *N. tabacum roq1-1* mutant was generated using a CRISPR/CAS9 construct targeting exon 1 of the *Roq1* gene (Supplementary Figure S6). Both RipB alleles triggered a strong hypersensitive/cell death response in wild-type *N. tabacum*, indicating immune activation. This response was absent in the *roq1-1* mutant but could be restored by transiently expressing Roq1 along with XopQ, RipB_GMI__1000_, or RipB_Molk__2_ (Figure 4).

### *Roq1* Tomatoes Are Resistant to *Ralstonia* Containing RipB

**FIGURE 5:**
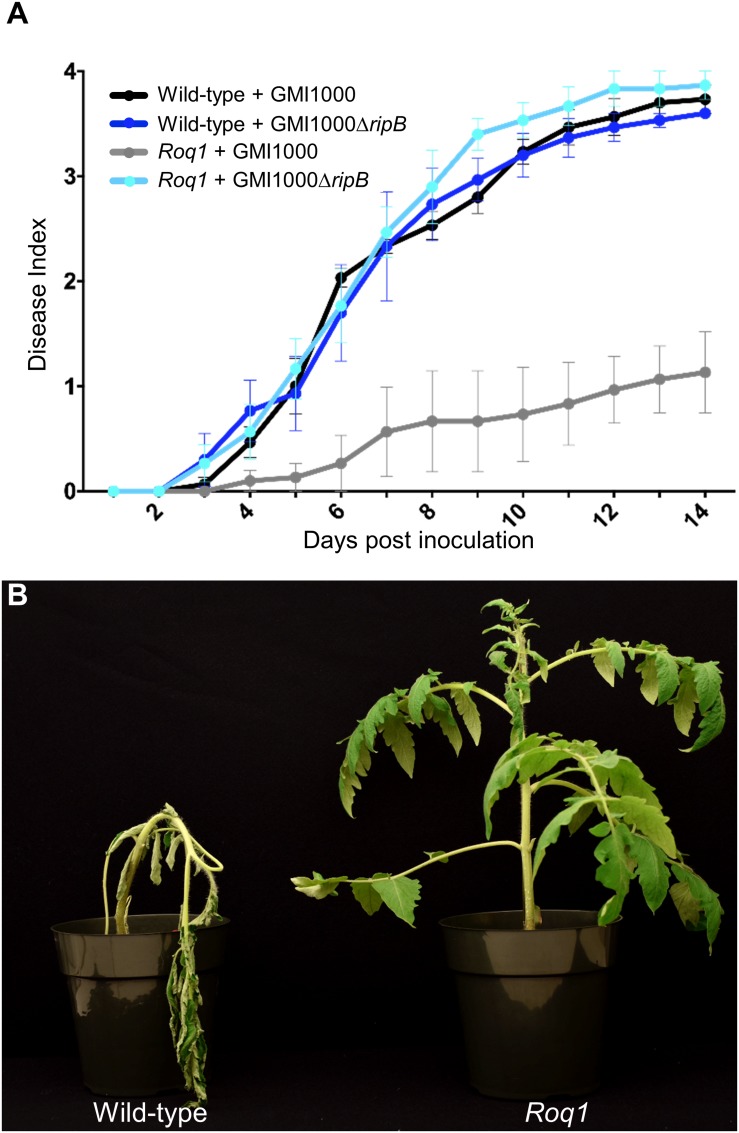
Bacterial Wilt disease development in Roq1 tomatoes. **(A)** Wild-type and *Roq1* tomato plants were infected with wild-type and RipB mutant *Ralstonia* strain GMI1000 by soil soak inoculation. Disease symptoms were monitored over 14 days, with no wilting corresponding to a Disease Index of 0 and complete wilting corresponding to a Disease Index of 4. Error bars indicate standard error from four biological replicates. Disease progression of wild-type *Ralstonia* on tomatoes expressing *Roq1* was significantly lower (*p* < 0.05) than the three other conditions (Friedman test with Dunn’s multiple comparisons test). **(B)** Wild-type and *Roq1* tomato plants 8 days after petiole inoculation with approximately 2000 cells of wild-type GMI1000.

Our observation that Roq1 can recognize RipB in leaf transient expression assays suggested that Roq1 can mediate resistance to bacterial wilt caused by *Ralstonia* in tomato. We tested this hypothesis by challenging wild-type and *Roq1*-expressing tomato plants with *Ralstonia* strain GMI1000 using a soil soak inoculation disease assay. Wild-type plants developed severe wilting approximately 7 days after inoculation, whereas *Roq1* tomato plants remained mostly healthy over the 2-week time course (Figure 5A and Supplementary Figure S7). The *Roq1* tomato plants were susceptible to a deletion mutant lacking RipB (GMI1000 Δ*ripB*). We also challenged plants by introducing bacteria directly to the xylem by placing bacteria on the surface of a cut petiole. Wild-type plants were wilted by eight days whereas *Roq1* plants remained healthy (Figure 5B). Tomatoes expressing *Roq1* were also resistant to *Ralstonia* strain UW551, which is a race 3 biovar 2 potato brown rot strain from phylotype II that has a RipB allele (Figure 3 and Supplementary Figure S8).

### Distribution of RipB Alleles in *Ralstonia*

To investigate the potential for using *Roq1* to protect plants from *Ralstonia*, we investigated the occurrence of RipB alleles in select strains. Table 2 lists some *Ralstonia* strains with their known hosts along with their respective phylotype and RipB allele accession information. Table 2 illustrates that strains lacking putative functional RipB alleles correlate with strains that are virulent on tobacco, which contains a native *Roq1* gene. All strains in Table 2 except for tobacco pathogenic strains K60, Y45, BK1002, and OE1-1 contain putative full-length and functional RipB alleles. Relative to other RipB alleles, the K60 RipB allele is truncated after residue 437 and missing approximately 65 C-terminal residues and the OE1-1 allele is truncated after residue 417, missing approximately 77 residues based on a published genome sequence (Hayes et al., 2017) (Supplementary Figure S5). Y45 does not have a predicted RipB allele based on a draft genome sequence (Li et al., 2011). Published gene models for RipB disagree on which start codon is the correct one, leading some RipB alleles to look like they are missing part of the N terminus or have N-terminal extensions. Analysis of the DNA sequence of diverse RipB alleles showed that out of three possible in-frame start codons, only a single putative start codon is conserved among *Ralstonia* strains from all four phylotypes (Supplementary Figure S9), suggesting that this is the true start codon and eliminating the N-terminal discrepancy between different RipB alleles.

**TABLE 2:** RipB occurrence and host range in *Ralstonia*.

***Ralstonia*strain**	**Host(s)**	**Origin**	**Phylotype**	**RipB allele**	**RipB accession**
GMI1000	Tomato, Pepper, Arabidopsis	French Guyana	I	Present	CAD13773.2
RS1000	Tomato	Japan	I	Present	BAD42390.1
OE1-1	Tobacco	Japan	I	Truncated	RSOE_09080**
BK1002	Tobacco	Japan	not available	Truncated	BBI29704.1
Y45	Tobacco	China	I	Absent	None
K60	Tomato, Tobacco	United States	IIA	Truncated	B7R77_07450**
CFBP2957	Tomato	Martinique	IIA	Present	CBJ44424.1
Po82	Tomato, Banana, Potato	Mexico	IIB	Present	WP_014618158.1
IPO1609*	Potato	Netherlands	IIB	Present	WP_020956993.1
MolK2	Banana	Philippines	IIB	Present	WP_003278485.1
UW551*	Geranium	Kenya	IIB	Present	WP_020956993.1
CMR15	Tomato	Cameroon	III	Present	WP_020749919.1
PSI07	Tomato	Indonesia	IV	Present	WP_013213770.1
BDB R229	Banana	Indonesia	IV	Present	WP_078222314.1
R24	Clove	Indonesia	IV	Present	CCA88514.1

The published host range of select *Ralstonia* strains is listed along with the identified RipB allele. Truncated indicates that the identified allele is missing conserved residues at the C terminus and is putatively non-functional. * Indicates a race 3 biovar 2 strain. ** Indicates NCBI locus tag from genome accession CP009764.1 for strain OE1-1 and genome NCTK01000001.1 for strain K60.

## Discussion

*Roq1* expression in tomato confers strong resistance to *X. perforans*, *X. euvesicatoria*, *and P. syringae* pv. *tomato*. Its effectiveness is dependent on the presence of the recognized effector protein XopQ/HopQ1 (Figures 1, 2). Field trials revealed that tomatoes expressing *Roq1* were less susceptible to *X. perforans* than wild-type tomatoes in conditions approximating commercial production (Table 1). *Roq1* conferred a similar level of resistance as the *Bs2*-containing resistant check variety in one season and was slightly weaker in the other two. Bacterial spot caused by *X. perforans* can cause lesions on fruits, making them unsuitable for commercial sale, and also reduce plant productivity by damaging leaf tissue. The onset of fruit lesions requires high disease pressure during a particular phase of fruit development. Environmental conditions did not favor the development of fruit lesions and we did not observe significant fruit lesion formation on any of the genotypes in any of the three seasons. Despite showing a strong reduction in foliar disease symptoms, the *Roq1* line did not have a significantly greater yield than the susceptible parental variety. A possible explanation for this finding is that bacterial spot did not appear to be a major constraint on yield in any of the three seasons. In spring 2018, weather conditions promoted the development of disease only late in the season after much of the yield was already set. Fall 2018 was unseasonably hot and dry for most of the season resulting in low disease pressure and very poor yield for all genotypes. Of the three seasons, spring 2019 had weather conditions expected to be most conducive for observing an impact of bacterial spot on marketable yield with mid-season rain promoting the development of disease symptoms. The average marketable yield for the *Roq1* tomatoes was 27% higher than wild-type in this season, although a relatively small sample size (four replicate plots of 10 plants each) and a large variability of yield between plots resulted in a *p*-value of 0.08 by Student’s *t*-test. Notably the yield of the resistant check variety expressing *Bs2* was not significantly higher than the susceptible control in this season, though it was previously reported to give a yield increase of 1.5–11x relative to susceptible varieties under high disease pressure (Horvath et al., 2012). This suggests that bacterial spot was not severe enough to have a strong impact on yield in this season and that *Roq1* may result in an increase in marketable yield under stronger disease pressure.

It was unclear if *Roq1* could confer resistance to *Ralstonia* because it colonizes different tissues than *Xanthomonas* and *P. syringae*. While *Xanthomonas* and *P. syringae* colonize tomato leaf tissue, *Ralstonia* enters through the roots and colonizes xylem vessels. Although the type III secretion system is essential for virulence in *Ralstonia*, it is not clear when and where the pathogen delivers effectors into host cells. It was therefore not clear if *Roq1* would be able to confer resistance to this pathogen in tomato. Here we demonstrated that tomato plants expressing *Roq1* had strong resistance to *Ralstonia* expressing RipB as measured by both soil soak and cut-petiole inoculation assays (Figure 5). In addition to conferring resistance to the phylotype I strain GMI1000, *Roq1* also confers resistance to *Ralstonia* race 3 biovar 3 strain UW551, a phylotype II strain that can overcome other known sources of bacterial wilt resistance in tomato (Milling et al., 2011). Some but not all of the *Roq1* tomatoes inoculated with GMI1000 by soil soak were colonized by a moderate or low population of *Ralstonia* (Supplementary Figure S10). This observation suggests that Roq1-mediated immune responses may act to both restrict the establishment of vascular colonization and separately reduce bacterial titers if colonization does occur. Activation of immune receptors, including Roq1, is known to induce many defense-associated genes with different putative activities (Sohn et al., 2014; Qi et al., 2018), presumably acting to inhibit pathogen virulence by distinct mechanisms. The observation that Roq1 inhibits both colonization establishment and population growth suggests that at least two independent downstream defense responses mediate the observed resistance phenotype.

The *Roq1* tomatoes were susceptible to a *Ralstonia* mutant lacking *RipB*, indicating that the resistance depends on the interaction between RipB and Roq1. This is consistent with the observation that several naturally occurring *Ralstonia* strains that can infect tobacco have a truncated or are missing the RipB effector (Table 2) (Nakano and Mukaihara, 2019), suggesting that losing RipB can allow the pathogen to overcome the native *Roq1* gene present in *N. tabacum*. Tobacco-infecting strains K60 and OE1-1 contain independently truncated RipB alleles (Figure 3 and Supplementary Figure S5) and there have likely been multiple independent gene loss events which enable strains to evade Roq1-mediated resistance. Similarly, HopQ1 has been lost in strains of *P. syringae* that can infect tobacco (Denny, 2006; Ferrante and Scortichini, 2009; Li et al., 2011). This suggests that this effector is not essential for virulence in all circumstances and it would therefore be prudent to deploy *Roq1* in combination with other disease resistance traits to avoid resistance breakdown due to pathogens losing XopQ/HopQ1/RipB. Although minor foliar symptoms were observed on the *Roq1* tomatoes, particularly in spring 2019 (Table 1), we do not believe this was due to a naturally occurring XopQ mutant arising during the trial. Instead, we think that these low disease scores may have been the result of fungal diseases, which can cause foliar symptoms that look similar to bacterial spot, or by the *Roq1* tomatoes supporting a low level of bacterial growth.

No other known NLR immune receptor confers resistance against such a broad range of bacterial pathogenic genera as Roq1. Effectors that are recognized by NLR proteins act as avirulence factors and are under strong evolutionary pressure to diversify or be lost to evade immune activation. Therefore the effector repertoires of pathogens are often quite diverse, with relatively few “core” effectors conserved within a species and even fewer shared between different genera (Grant et al., 2006). Effectors recognized by plant NLRs are typically narrowly conserved within a single bacterial genus (Kapos et al., 2019). One such effector is AvrBs2, recognized by the *Bs2* receptor from pepper, which is present in many *Xanthomonas* strains but is absent from *P. syringae* and *Ralstonia*. In contrast, XopQ/HopQ1/RipB is highly conserved in most *Xanthomonas*, *P. syringae*, and *Ralstonia* strains that cause disease in crop plants including kiwi (*P. syringae* pv. *actinidae*), banana (*Ralstonia* and *X. campestris* pv. *musacearum*), stone fruit (*P. syringae*), pepper (*X. euvesicatoria*), citrus (*X. citri*), strawberry (*X. fragariae*), brassica (*X. campestris*), rice (*X. oryzae*), potato (*Ralstonia*), and others. *Ralstonia* race 3 biovar 2 strains are of particular concern because they are cold tolerant and potentially threaten potato cultivation in temperate climates. As a result, *Ralstonia* race 3 biovar 2 strains are strictly regulated quarantine pathogens in Europe and North America and is on the United States Select Agent list. The ability of *Roq1* to protect tomato from the race 3 biovar 2 strain UW551 (Supplementary Figure S8) suggests that *Roq1* can also protect potato from this high-concern pathogen. This work demonstrates the widespread potential of using naturally occurring plant immune receptors to safely, sustainably, and economically manage diverse and difficult to control pathogen species.

## Data Availability Statement

All datasets generated for this study are included in the article/Supplementary Material.

## Author Contributions

NT and AS wrote the manuscript and performed *Pseudomonas* and *Ralstonia* petiole infection assays. AS carried out *Xanthomonas* infection and *Agrobacterium* transient expression experiments. UG and SH performed *Xanthomonas* field experiments. CH constructed the *Ralstonia* knockout and performed *Ralstonia* soil soak assays, supervised by CA. All authors analyzed the results and edited and approved the manuscript.

## Conflict of Interest

AS and NT are employees of and have a financial stake in Fortiphyte Inc., which has intellectual property rights related to the Roq1 resistance gene. The remaining authors declare that the research was conducted in the absence of any commercial or financial relationships that could be construed as a potential conflict of interest.
