# Supplementary material for: The Immune Receptor Roq1 Confers Resistance to the Bacterial Pathogens Xanthomonas, Pseudomonas syringae, and Ralstonia in Tomato
Source: Front Plant Sci. 2020 Apr 23;11:463. doi: 10.3389/fpls.2020.00463 (PMC7192161; doi:10.3389/fpls.2020.00463)
Supplement: Supplementary file 1 [file Data_Sheet_1.pdf]

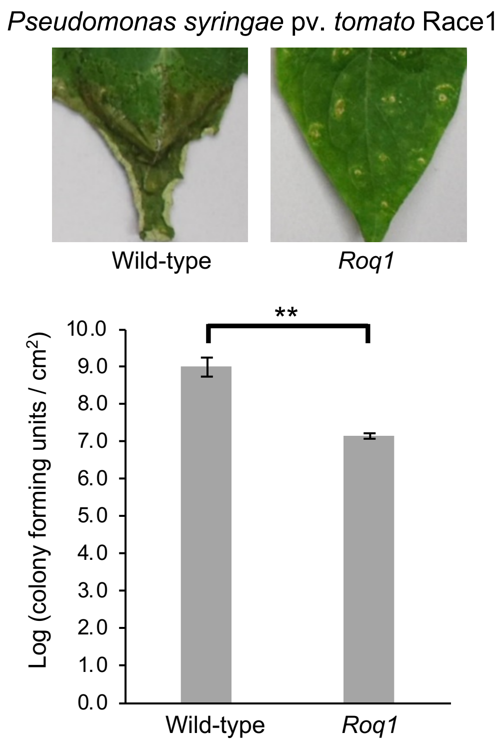

Supplementary Figure S1. Growth of *Pseudomonas syringae* pv. *tomato* Race 1 in wild-type and *Roq1* tomatoes. *Pseudomonas syringae* pv. *tomato* Race 1 was infiltrated into wild-type and *Roq1* tomatoes. At four days post infiltration disease symptoms were imaged (top) and colony forming units were determined by dilution plating of homogenized tissue. Error bars indicate standard deviation. ** = $p < 0.01$ by Student's t-test.

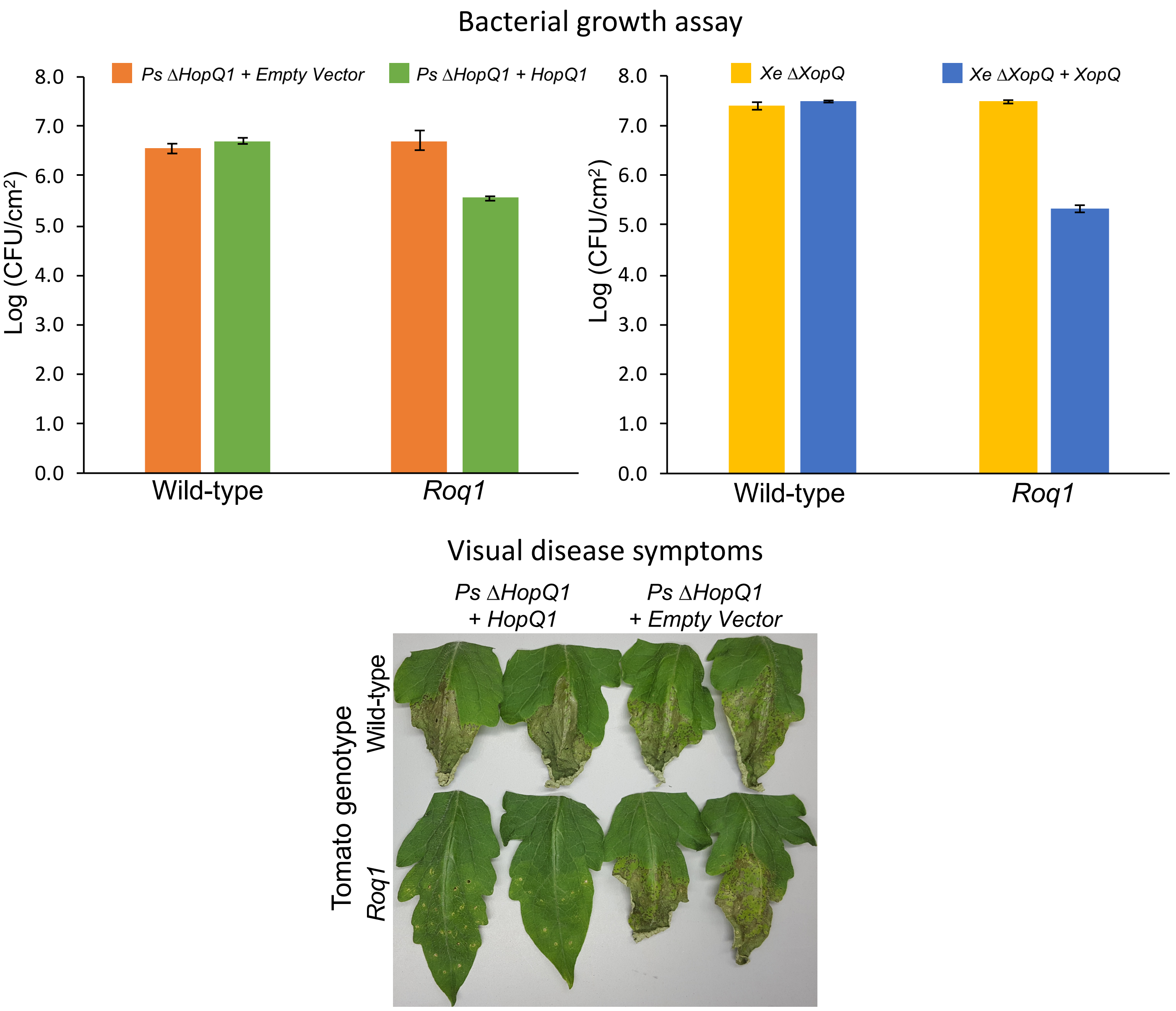

Supplementary Figure S2. Disease assay of complemented HopQ1/XopQ mutants. The indicated genotypes of *Pseudomonas syringae* strain DC3000 (*Ps*) and *Xanthomonas euvesicatoria* strain 85-10 (*Xe*) were infiltrated into leaf tissue of wild-type tomato and tomato expressing *Roq1* at a low density (OD_{600} = 0.00005 and 0.0001 respectively). Punches of leaf tissue were homogenized and plated to determine colony forming units (CFU) at four days post infiltration for *Pseudomonas syringae* and six days post infiltration for *Xanthomonas euvesicatoria*. The error bars show the standard deviation from three biological replicates per condition. The visual disease symptoms for *Pseudomonas syringae* were imaged at four days post infiltration.

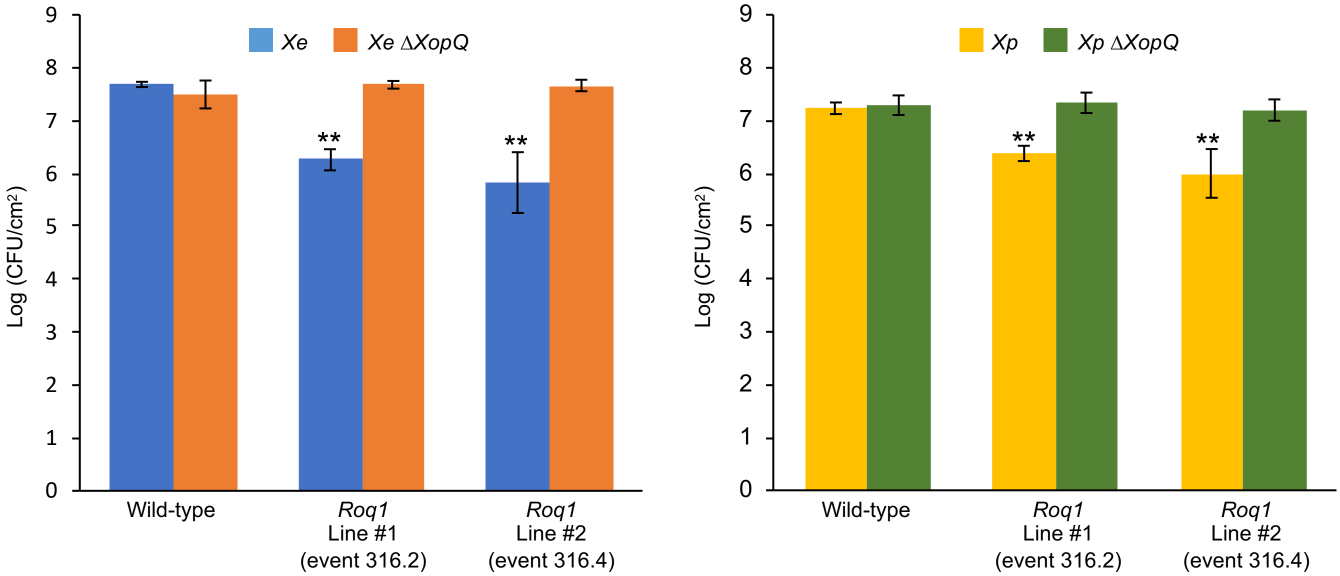

Supplementary Figure S3. **Resistance phenotype of independent tomato lines expressing *Roq1***. Wild-type and XopQ deletion mutants of *Xanthomonas euvesicatoria* 85-10 and *Xanthomonas perforans* 4B were infiltrated at a low inoculum (OD_{600} = 0.0001) into leaf tissue of wild-type tomatoes and two independent tomato lines expressing *Roq1*. Six days post infiltration, the leaf tissue was homogenized and plated to quantify bacterial abundance by colony forming units (CFU). Error bars indicate standard deviation from six replicates for each condition. ** indicates p-value < 0.01 in comparison to the wild-type by Student's t-test. *Roq1* line #2 was selected for further characterization and use in field trials.

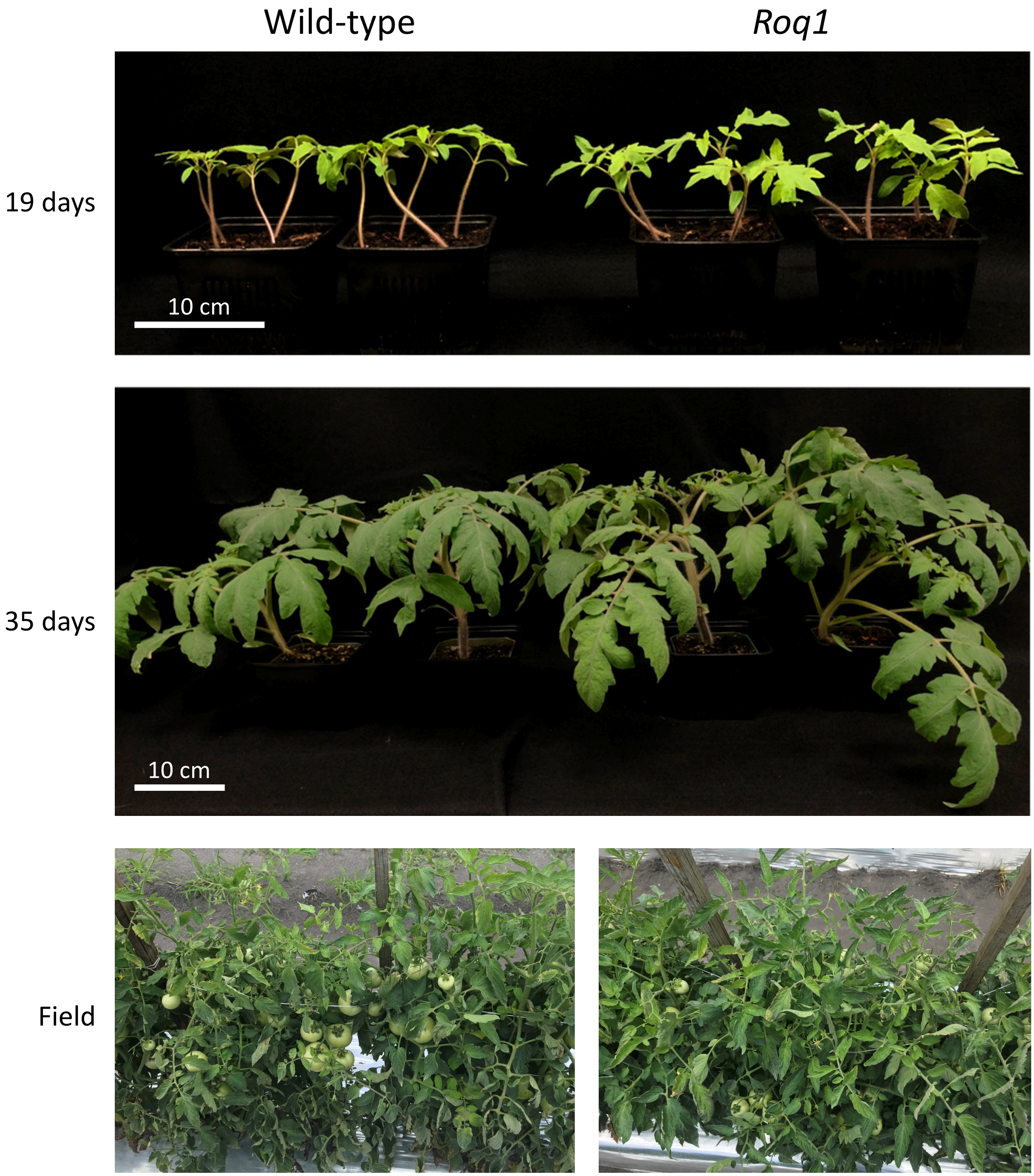

Supplementary Figure S4. Growth phenotype of *Roq1* tomato plants under no and low disease pressure. Images of 19-day old and 35-day old growth chamber grown, and field grown wild-type Fla. 8000 and *Roq1* tomatoes show no obvious stunting, necrosis, or other growth defects in the *Roq1* tomatoes. Although some foliar disease symptoms are visible for the field grown wild-type tomatoes here, the plants were under low disease pressure at this stage due to the lack of warm and rainy conditions favoring disease.

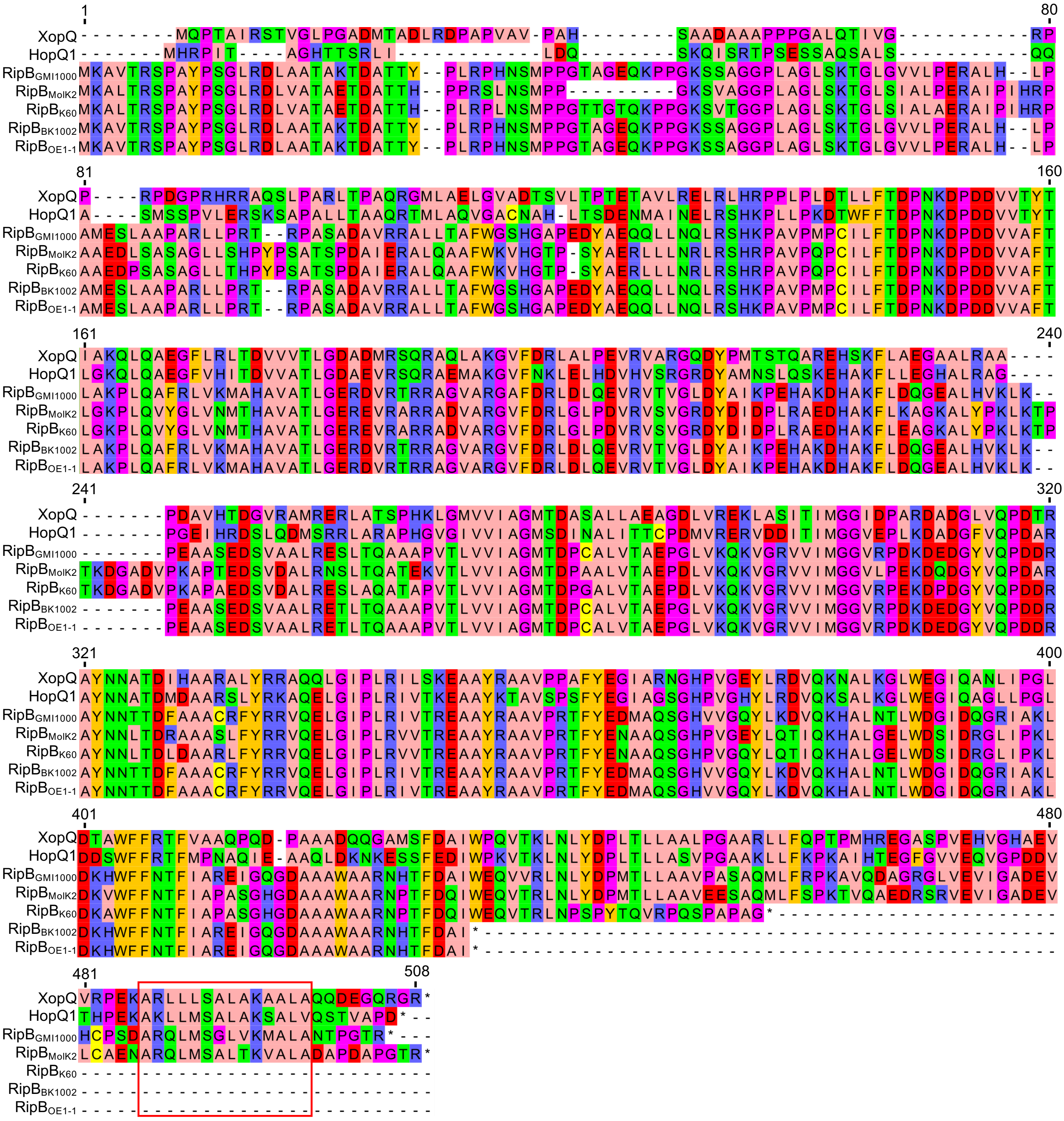

Supplementary Figure S5. Protein alignment of XopQ, HopQ1 and RipB. Protein sequences from *Xanthomonas euvesicatoria* 85-10 (XopQ), *Pseudomonas syringae* DC3000 (HopQ1), and the *Ralstonia* strains GMI1000, MolK2, K60, and BK1002 (RipB) were used to generate the alignment using ClustalO. The putative full length BK1002 RipB sequence based on analysis shown in Supplemental Figure S 9 was used for this alignment and differs from the NCBI accession BBI29704.1. The boxed region indicates a motif that is conserved in XopQ, HopQ1 and the GMI1000 and MolK2 alleles of RipB but absent in the putatively truncated K60, BK1002 and OE1-1 alleles.

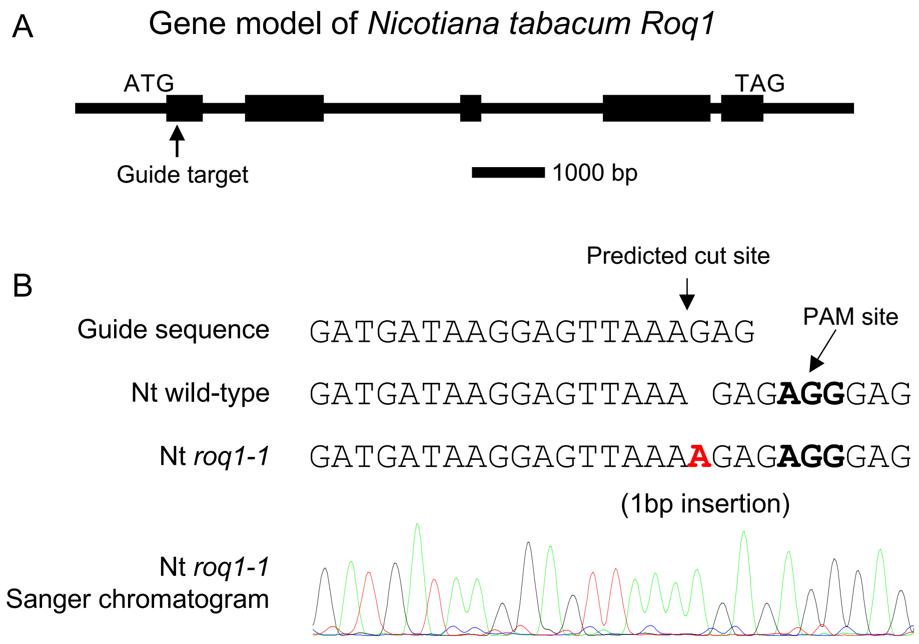

Supplementary Figure S6. **Sequence of *roq1 N. tabacum* mutants**. A CRISPR / CAS9 construct was transformed into *N. tabacum* with a guide targeting the first exon of the *Roq1* gene (A). Transformed plants were selected by resistance to kanamycin and then genotyped by PCR and Sanger sequencing to look for the presence of mutations at the target site. A mutant containing a single base pair A insertion at the predicted cut site was identified and named Nt *roq1-1* (B). The target sequence of this guide is conserved between *N. tabacum* and *N. benthamiana* and was also used for the generation of *N. benthamiana roq1* mutants published in Qi et al. 2018.

Wild-type *Roq1*

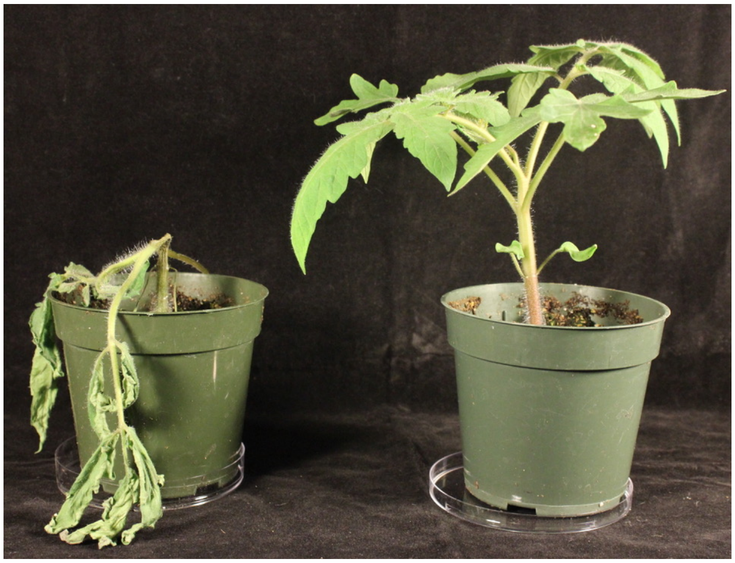

Supplementary Figure S7. *Ralstonia* disease symptoms from soil soak inoculation. This image shows representative plants from the *Ralstonia* disease assay depicted in Figure S 5A. The plants were infected with wild-type *Ralstonia* strain GMI1000 by soil soak with a 50 mL solution containing $1x10^8$ colony forming unit / mL. The plants were imaged 8 days post inoculation at an age of 25 days. The wild-type tomato plants exhibited severe wilting (left) whereas the *Roq1* tomatoes showed no or minor disease symptoms (right).

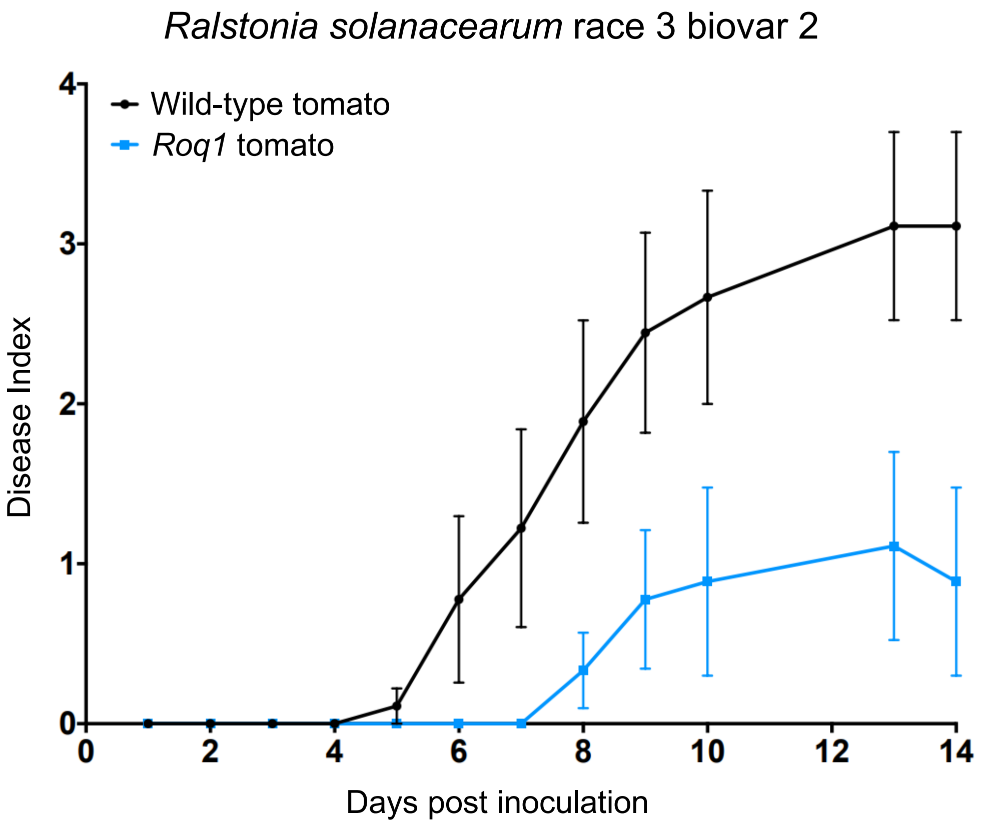

Supplementary Figure S8. Disease assay with *Ralstonia solanacearum* race 3 biovar 2. Wild-type tomato plants (cv. Fla. 8000) and tomato plants expressing *Roq1* were soil-soak inoculated with *R. solanacearum* race 3 biovar 2 strain UW551. Disease symptoms were scored over the course of two weeks, with a Disease Index of 0 corresponding to no symptoms and a Disease Index of 4 corresponding to complete wilting. Error bars indicate standard error from three biological replicates.

**GMI1000**											**M**	**K**	**A**	**V**	**T**	**R**	**S**	**P**	**A**	**Y**	**P**	**S**	**G**	**L**	**R**
**GMI1000**	GCC	CGA	CCG	GCA	ACT	CGT	CAG	ACT	ATG	GCG	ATG	AAG	GCC	GTC	ACC	CGA	TCA	CCA	GCC	TAT	CCG	TCC	GGC	CTA	CGC
**BK1002**	GCC	CGA	CCG	GCA	ACT	CGT	CAG	ACT	ATG	GCG	ATG	AAG	GCC	GTC	ACC	CGA	TCA	CCA	GCC	TAT	CCG	TCC	GGC	CTA	CGC
**UW551**	GCC	CGG	CCG	GCA	AGT	CAT	CAG	ACT	AGG	GCG	ATG	AAG	GCC	CTC	ACC	CGA	TCC	CCC	GCT	TAT	CCG	TCC	GGT	CTG	CGC
**R24**	ACA	CGG	CTC	GCA	GCC	CGC	CAG	ACT	ACG	GCG	ATG	AAA	GCC	GCC	CCC	CGA	TCG	CCA	GCC	GAT	CCG	TCC	AGC	CTG	CCC
**CMR15**	ATC	CGA	TCG	GCG	GCC	CGC	CAG	ACT	GCG	GCG	ATG	AAA	GCC	GTC	ACC	CGA	TCG	TCA	GTC	TAT	TCA	CCC	GGC	CCG	GTT

**GMI1000**	**D**	**L**	**A**	**A**	**T**	**A**	**K**	**T**	**D**	**A**	**T**	**T**	**Y**	**P**	**L**	**R**	**P**	**H**	**N**	**S**	**M**	**P**	**P**	**G**	**T**
**GMI1000**	GAC	CTT	GCG	GCC	ACT	GCC	AAG	ACG	GAT	GCC	ACA	ACG	TAC	CCG	CTG	CGA	CCA	CAC	AAC	AGC	ATG	CCG	CCA	GGC	ACA
**BK1002**	GAC	CTT	GCG	GCC	ACT	GCC	AAG	ACG	GAT	GCC	ACA	ACG	TAC	CCG	CTG	CGA	CCA	CAC	AAC	AGC	ATG	CCG	CCA	GGC	ACA
**UW551**	GAC	CTT	GTG	GCC	ACT	GCC	GAG	ACG	GAT	GCC	ACA	ACG	CAC	CCG	CCG	CGA	TCG	CTC	AAC	AGC	ATG	CCG	CCC	GGC	AAA
**R24**	GAC	CTT	GCA	GCC	ACT	GCC	GAG	ACG	GAT	GCC	ACG	AAT	CAC	CCG	TTG	CGA	CCA	CAC	AGC	AAC	AAG	CCG	CCA	AGC	ACA
**CMR15**	GGC	CTT	GCC	GCC	GCG	ACC	GAT	ACG	GAC	ACC	ACG	AAC	CGC	CGG	CCC	GAA	CCG	CGC	GGC	AAC	ATG	TCG	CCC	GGC	AAA
PSI07																			AGC	AAC	AAG	CCG	CCA	GGC	
BDB A2-HR MARDI																			AGC	AAC	AAG	CCG	CCA	GGC	

**Strain**	**Phylotype**	**NCBI accession**
GMI1000	I	CAD13773.2
BK1002	I	LC459955.1
UW551	II	NCTI00000000.1
R24	IV	CCA88514.1
CMR15	III	WP_020749919.1
PSI07	IV	WP_013213770.1
BDB A2-HR MARDI	IV	WP_078222314.1

Supplementary Figure S9. Putative start codon annotation of RipB. The N-terminal amino acids and codons of GMI1000 RipB were aligned to the DNA sequence of other RipB alleles. In-frame start codons are boxed, with the green box indicating the start codon that is conserved across RipB accessions from all four *Ralstonia* phylotypes. The start codons boxed in orange have been annotated for some RipB accessions but are not conserved across all the phylotypes and may therefore be incorrect. Nucleotide polymorphisms relative to GMI1000 are shown in bold, with synonymous changes in green and non-synonymous changes in red. Notably the phylotype IV strains R24, PSI07 and BDB A2-HR MARDI (for which only six codons are shown) are all lacking the downstream start codon.

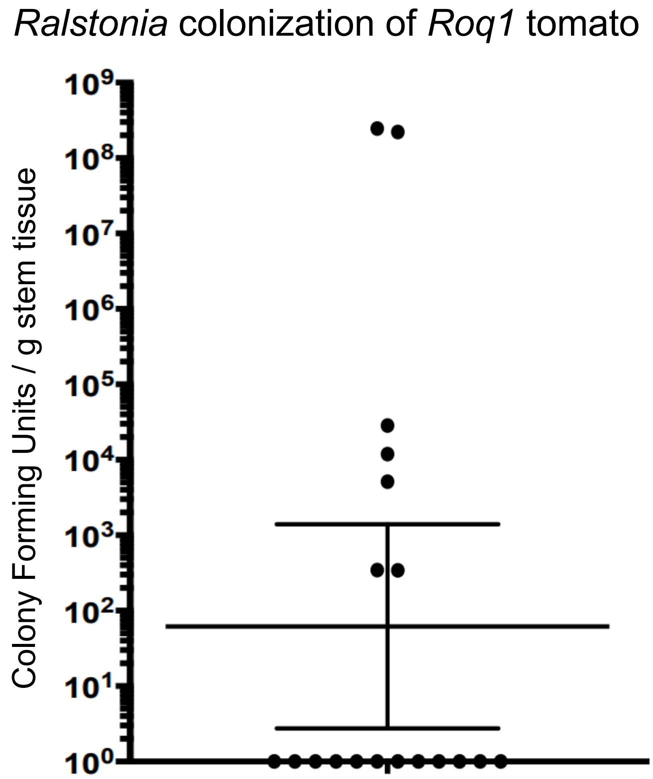

Supplementary Figure S10. Colonization of *Roq1* tomato plants following inoculation with *Ralstonia*. Tomato plants expressing *Roq1* were infected with *Ralstonia* strain GMI1000 using the soil soak method. After two weeks all wild-type tomato plants had wilted but nearly all the *Roq1* tomato plants appeared healthy with no or minimal disease symptoms (Figure S 5A). Bacterial colonization in the *Roq1* plants were measured by homogenizing and dilution plating mid-stem sections to determine colony forming units per gram stem tissue. Out of 19 plants tested from three independent biological replicates, twelve had no detectable colonization (limit of detection = 100 colony forming units / gram stem tissue), five had low colonization ($<1x10^5$) and two had moderate colonization ($\sim 2x10^8$). The wild-type tomatoes were dead and unable to be assayed at this timepoint, but susceptible tomato plants typically reach colonization densities of 10^9 or 10^{10} CFU / g stem tissue (Lowe-Power et al., 2018; Zhang et al., 2018; Zhang et al., 2019). All the *Roq1* tomato plants in this assay had a Disease Index of 0 except for one plant which had a Disease Index of 3 and a colonization of 2.2×10^8.

Name	Sequence	Description
AS-940	TCCTAAGCTTTAGGGGAGAA	roq1-1 genotyping forward
AS-941	AAAAATGACCACTGACCCAT	roq1-1 genotyping reverse
AS-946	TGGTCTCC GAGC ATGTTGACTTCATCTTCC	NbRoq1 CDS part 1 forward
AS-947	TGGTCTCC GACT TCCACCAATGCATCa	NbRoq1 CDS part 1 reverse
AS-973	TGGTCTCC AGTC CATCCATCTGTTGGGTTTCT	NbRoq1 CDS part 2 forward
AS-951	TGGTCTCC TTGG CTATCTGTTTATGAGCATTTCG	NbRoq1 CDS part 2 reverse
AS-968	GAAAGACAACTGCTGCAAG	Sequencing NbRoq1
AS-969	AGAGGACAAAATCCAAATGC	Sequencing NbRoq1
AS-970	CCTAGTAGTATTTGGAGATTCAGA	Sequencing NbRoq1
AS-530	accggatctagaaggccttg	Sequencing inserts in pORE E4
AS-159	TCCGTCCAAAAGAAAATAAA	Sequencing inserts in pORE E4
AS-531	accggcaacaggattcaat	Sequencing inserts in pORE E4
ripBupF	taaaacgacggccagtgccaCCGACAAGACGACCATCTC	RipB upstream forward
ripBupR	ccggcgtgttCGCCATAGTCTGACGAGTTG	RipB upstream reverse
ripBdwnF	gactatggcgAACACGCCGGGTACGCGC	RipB downstream forward
ripBdwnR	cagctatgaccatgattacgGCATGCACTTCTTCAACCCGGTG	RipB downstream reverse

Supplementary Table S1. Sequence of oligonucleotide primers used in this research**,** listed in 5’ to 3’ orientation. Note that AS-941 was designed for genotyping the *Nicotiana benthamiana* allele and has a 1 bp mismatch relative to *Nicotiana tabacum* but is still functional.
